# Supplementary material for: The Systems Biology Research Tool: evolvable open-source software
Source: BMC Syst Biol. 2008 Jun 29;2:55. doi: 10.1186/1752-0509-2-55 (PMC2446383; doi:10.1186/1752-0509-2-55)
Supplement: Additional file 1 — SBRT Archive. An archive of the current version of the Systems Biology Research Tool. [file 1752-0509-2-55-S1.zip › sbrt-1.4.0/doc/developers_guide/api/sbrt/shell/text/chem/package-tree.html]

sbrt.shell.text.chem Class Hierarchy


|  |  |  |
| --- | --- | --- |
| **Overview** \| **Package** \| Class \| Use \| **Tree** \| **Deprecated** \| **Index** \| **Help** | |  |
| **PREV**   **NEXT** | **FRAMES**    **NO FRAMES**     **All Classes** |  |


---


## Hierarchy For Package sbrt.shell.text.chem

**Package Hierarchies:**: All Packages

---

## Class Hierarchy

- java.lang.Object
  - sbrt.shell.text.chem.**CatalystFormatV1** (implements sbrt.shell.text.chem.CatalystFormat)- sbrt.shell.text.chem.**CatalystFormatV2** (implements sbrt.shell.text.chem.CatalystFormat)- sbrt.shell.text.chem.**ChemSpeciesFormatV1** (implements sbrt.shell.text.chem.ChemSpeciesFormat)- sbrt.shell.text.chem.**IrrevRxnFormatV1** (implements sbrt.shell.text.SimpleFormat<T>)- sbrt.shell.text.chem.**IrrevRxnFormatV2** (implements sbrt.shell.text.SimpleFormat<T>)- sbrt.shell.text.chem.**RevRxnFormatV1** (implements sbrt.shell.text.SimpleFormat<T>)- sbrt.shell.text.chem.**RevRxnFormatV2** (implements sbrt.shell.text.SimpleFormat<T>)- sbrt.shell.text.chem.**RxnNameFormatV1** (implements sbrt.shell.text.chem.RxnNameFormat<T>)

## Interface Hierarchy

- sbrt.shell.text.**Format**
  - sbrt.shell.text.**SimpleFormat**<T> (also extends sbrt.shell.text.Formatter<T>, sbrt.shell.text.Parser<T>)
    - sbrt.shell.text.chem.**CatalystFormat**- sbrt.shell.text.chem.**ChemSpeciesFormat**- sbrt.shell.text.chem.**RxnNameFormat**<T>
  - sbrt.shell.text.**Formatter**<T>
    - sbrt.shell.text.**SimpleFormat**<T> (also extends sbrt.shell.text.Format, sbrt.shell.text.Parser<T>)
      - sbrt.shell.text.chem.**CatalystFormat**- sbrt.shell.text.chem.**ChemSpeciesFormat**- sbrt.shell.text.chem.**RxnNameFormat**<T>
    - sbrt.shell.text.**Parser**<T>
      - sbrt.shell.text.**SimpleFormat**<T> (also extends sbrt.shell.text.Format, sbrt.shell.text.Formatter<T>)
        - sbrt.shell.text.chem.**CatalystFormat**- sbrt.shell.text.chem.**ChemSpeciesFormat**- sbrt.shell.text.chem.**RxnNameFormat**<T>

---


|  |  |  |
| --- | --- | --- |
| **Overview** \| **Package** \| Class \| Use \| **Tree** \| **Deprecated** \| **Index** \| **Help** | |  |
| **PREV**   **NEXT** | **FRAMES**    **NO FRAMES**     **All Classes** |  |


---
